# A blast from the past: *ROS1* on the brain

**DOI:** 10.18632/oncotarget.26752

**Published:** 2019-03-01

**Authors:** Igor Odintsov, Romel Somwar, Monika A. Davare

**Affiliations:** Department of Pediatrics, Oregon Health Sciences University, Beaverton, OR, USA

**Keywords:** cancer, glioma, ROS1, kinase, inhibitor

Gliomas are the most common central nervous system (CNS) tumors affecting 6.6 per 100,000 patients in the United States [1]. This umbrella term covers a heterogeneous group of neoplasms believed to arise from glial cells, with glioblastoma (GBM) representing the most common (half of newly diagnosed gliomas) and most aggressive subtype. Multimodal treatment of GBM with a combination of surgery, radiation and chemotherapy achieves a dismal 5-year survival in about 5% of GBM patients [1]. Therapy aimed at specific molecular alterations holds the potential to improve clinical outcomes for GBM patients.

To identify tyrosine kinase gene fusions in gliomas we interrogated The Cancer Genome Atlas (TCGA) GBM dataset using a bioinformatics algorithm that uses RNA-sequencing data and evaluates 5′–3′ gene expression imbalance. We found intrachromosomal microdeletions in 6q22 that produce *ROS1* gene fusions, *GOPC/ROS1* and *CEP85L/ROS1.* Their occurrence was validated in independent datasets from MSKCC IMPACT and Foundation Medicine. Of importance, *ROS1* fusion expression was mutually exclusive of *EGFR, PDGFRA,* and *IDH1* alterations. Interestingly, *ROS1* rearranged tumors were enriched in homozygous *CDKN2A/B* deletions: 9 of 10 fusion-positive GBM samples compared to non-small cell lung cancer where only 3 of 28 samples exhibited CDKN2A/B loss (MSK-IMPACT sequencing dataset interrogated *via* cbioportal.org). In addition, ROS1 fusion-positive GBMs harbored concurrent PTEN aberrations (7 of 10 cases). Charest et al. showed that GOPC-ROS1 cooperates with homozygous loss of pl6Ink4a/pl9Arf (murine orthologues of *CDKN2A/B)* to form highly penetrant brain tumors that histologically resemble high-grade gliomas [2]. Further, they demonstrated activation of the MTOR kinase pathway and corresponding sensitivity to the MTOR inhibitor, rapamycin. We postulate that *PTEN* mutations result in activation of PI3K/AKT/MTOR signaling axes and synergize with GOPC-ROS1. Taken together this suggests a cooperative driver role of *ROS1* fusions in glioma.

Of note, *ROS1* rearrangements were missed in the original TCGA publications. Glioma-enriched *ROS1* fusions, such as GOPC-ROS1, are generated from relatively small deletions between two proximal genes. Short distances between fusion partners can preclude detection by clinical fluorescence in situ hybridization, unless specific probe sets designed to detect this fusion are used. Importantly, bioinformatic analyses can miss some rearrangements due to poor read depth over chimeric junctions. However, newer fusion finding algorithms have improved fusion gene detection. *FGFR/TACC* fusions were also identified in GBM by re-examining already published sequencing data [3], highlighting the need to methodically evaluate 'omics data for identification of targetable gene rearrangements.

*ROS1,* encoding an orphan tyrosine kinase receptor, was originally isolated as a potential oncogene in 1984 when its transforming potential was demonstrated in NIH3T3 cells [4]. This putative oncogene, first called *MCF3,* was later renamed *ROS1* (originally *c-ros-1 or c-ROS)* due to its homology to the cloned v-ros sequence of avian UR2 sarcoma virus [5]. In 1987, expression screening of human cell lines showed elevated *ROS1* expression in a subset of glioma cell lines. Of particular interest was the U118MG GBM cell line with an aberrant configuration of the *ROS1* gene locus [6]. This rearrangement was later characterized as an intrachromosomal microdeletion leading to *FIG/ ROS1* fusion *(FIG* was renamed GO*PC).* Congruent with previous studies, we showed the transformative potential of *GOPC*/ROS1 fusion in multiple cellular lineages including in immortalized human astrocytes [7]. Multiple currently available ROS1 tyrosine kinase inhibitors (TKI) block the mitogenic effects of GOPC-ROS1, the recurrent ROS1 fusion in GBM [7].

Clinical response to a ROS1 TKI was first observed in *ROS1* fusion-positive lung adenocarcinoma patients. *ROS1* fusions are now recognized as dominant oncogenes in approximately 2% of lung adenocarcinomas and their therapeutic inhibition is one of the most promising recent developments in the field. Moreover, ROS1 TKIs such as lorlatinib and entrectinib have shown intracranial efficacy in brain metastasis [8, 9]. We showed that lorlatinib significantly reduces tumor growth in an orthotopic preclinical murine model of *GOPC/ROS1*-driven GBM. These studies warrant future clinical investigation of this therapeutic strategy for brain tumors driven by *ROS1* rearrangements. However, given that *ROS1* fusion-positive GEMs frequently harbor concurrent aberrations in genes that regulate cell cycle, cell growth or survival *(CDKN2A/B, PTEN),* monotherapy maybe insufficient for achieving a long-term durable response. We posit that to prevent or delay emergence of resistance, combination or metronomic treatment with ROS1 and respective signaling pathway inhibitors (e.g., trametinib: MEK; rapamycin: MTOR) may be required. Additional pre-clinical studies to assess the duration of response, profile resistance pathway and evaluate drug combination strategies are needed.

Our finding of *ROS1* rearrangements in 1 of the 5 studied pediatric glioblastomas urges future investigation to clarify the occurrence in this cohort. Even if this proportion is overstated, GBM poses a considerable clinical challenge in pediatric oncology, with a 5-year survival of pediatric patients less than 17% [1]. Recent publications show that *GOPC/ROS1* and *CEP85L/ROS1* gene fusions are also present in pediatric low-grade glioma and diffuse astrocytoma [10]. Of note, targeted therapy presents an even more promising concept in this age group, since conventional treatment modalities such as cranial radiotherapy and chemotherapy often have profound adverse effects on the development of children.

In addition to *ROS1* rearrangements, others have identified *BRAF, EGFR, FGFR, MET* and *NTRK* alterations as drivers in GBM (Figure 1). As such, they provide an accessible opportunity for precision medicine interventions. Table 1 shows clinical targeting possibility for these kinase fusions using either FDA-approved agents, or those in ongoing clinical trials; the majority of agents in this table have not been tested in GMB patients. However, as we have reported, NGS methods can be improved to more reliably identify gene rearrangements, e.g. *ROS1* and *FGFR.* It is possible that other gene fusions such as *NTRK1* and *BRAF* are under-reported. RNA-based diagnostic methods may provide more reliable insight into fusion oncogene expression. Identifying these patients will enable their inclusion into ongoing clinical trials. One concern regarding clinical impact of these findings is that the number of patients with *ROS1*-driven glioblastoma is extremely small (≤1%). We suggest that given this devastating prognosis, every effort to actualize the benefit of precision medicine to improve long term outcomes is essential, no matter how rare the patient population.

**Figure 1:**
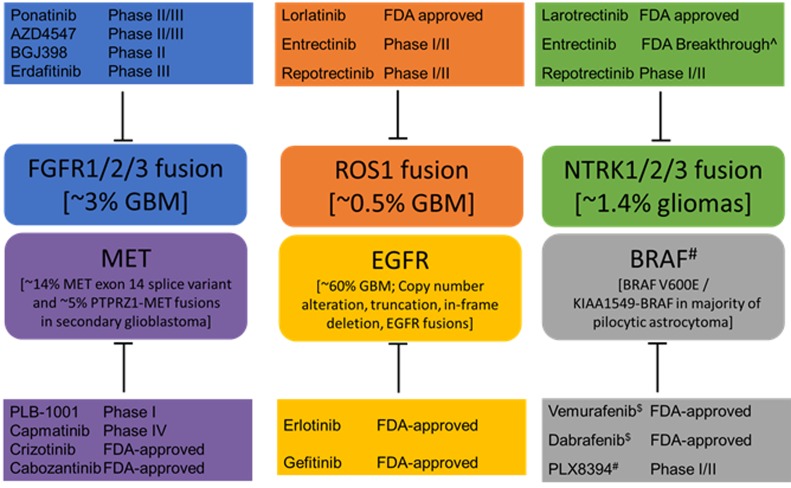
Genomic alterations identified in brain tumors and potential therapeutic agents The agents shown here have been tested in various cancer types and the highest stage of clinical trials or FDA approval for any type of tumor is indicated. Only PLB-1001 has been examined specifically in glioblastoma patients. Additional clinical studies are needed to assess the efficacy of many of these agents in the brain. ^$^ For BRAF V600E mutants. No current FDA-approved therapy for RAF fusions. ^#^ RAF drugs which block RAF dimerization are likely to act on fusions but clinical activity not published to date. ^Breakthrough designation indicates FDA signal to expedite the development given promising preliminary signs of clinical efficacy.

## References

[1] Ostrom QT (2016). Neuro Oncol.

[2] Charest A (2006). Cancer Res.

[3] Singh D (2012). Science.

[4] Fasano O (1984). Mol Cell Biol.

[5] Birchmeier C (1986). Mol Cell Biol.

[6] Birchmeier C (1987). Proc Natl Acad Sci U S A.

[7] Davare MA (2018). Clin Cancer Res.

[8] Shaw AT (2017). Lancet Oncol.

[9] Drilon A (2017). Cancer Discov.

[10] Johnson A (2017). Oncologist.

